# The Influence of Operating Pressure Oscillations on the Machined Surface Topography in Abrasive Water Jet Machining

**DOI:** 10.3390/ma18153570

**Published:** 2025-07-30

**Authors:** Dejan Ž. Veljković, Jelena Baralić, Predrag Janković, Nedeljko Dučić, Borislav Savković, Aleksandar Jovičić

**Affiliations:** 1 Faculty of Technical Sciences Čačak, University of Kragujevac, 32102 Cacak, Serbia; dejan.z.veljkovic@ftn.kg.ac.rs (D.Ž.V.); jelena.baralic@ftn.kg.ac.rs (J.B.); nedeljko.ducic@ftn.kg.ac.rs (N.D.); 2 Faculty of Mechanical Engineering, University of Niš, 18104 Niš, Serbia; predrag.jankovic@masfak.ni.ac.rs; 3 Faculty of Technical Sciences, University of Novi Sad, 21000 Novi Sad, Serbia

**Keywords:** abrasive water jet, pressure oscillations, machined surface, waviness

## Abstract

The aim of this study was to determine the connection between oscillations in operating pressure values and the appearance of various irregularities on machined surfaces. Such oscillations are a consequence of the high water pressure generated during abrasive water jet machining. Oscillations in the operating pressure values are periodic, namely due to the cyclic operation of the intensifier and the physical characteristics of water. One of the most common means of reducing this phenomenon is installing an attenuator in the hydraulic system or a phased intensifier system. The main hypothesis of this study was that the topography of a machined surface is directly influenced by the inability of the pressure accumulator to fully absorb water pressure oscillations. In this study, we monitored changes in hydraulic oil pressure values at the intensifier entrance and their connection with irregularities on the machined surface—such as waviness—when cutting aluminum AlMg3 of different thicknesses. Experimental research was conducted in order to establish this connection. Aluminum AlMg3 of different thicknesses—from 6 mm to 12 mm—was cut with different traverse speeds while hydraulic oil pressure values were monitored. The pressure signals thus obtained were analyzed by applying the fast Fourier transform (FFT) algorithm. We identified a single-sided pressure signal amplitude spectrum. The frequency axis can be transformed by multiplying inverse frequency data with traverse speed; in this way, a single-sided amplitude spectrum can be obtained, examined against the period in which striations are expected to appear (in millimeters). In the lower zone of the analyzed samples, striations are observed at intervals determined by the dominant hydraulic oil pressure harmonics, which are transferred to the operating pressure. In other words, we demonstrate how the machined surface topography is directly induced by water jet pressure frequency characteristics.

## 1. Introduction

With the appearance of new materials, machining problems have also arisen. High cutting forces or low clamping forces are required to machine new materials. Some materials must be machined so that high temperatures do not occur in the cutting zone. Due to all of the above, abrasive water jet machining is increasingly used for machining modern materials. Almost all types of materials can be cut with an abrasive water jet [1,2]. The great advantage of this technique is the very easy contour cutting, no matter how complex the contour. Conversely, the main limitations in the application of this technology are accuracy and quality. Today, machine parts with tolerances of less than 0.1 mm can be cut with an abrasive water jet [3]. The main disadvantage of this method is the appearance of increased roughness values on the machined surface, especially in the zone where the abrasive water jet exits the workpiece.

Also, an abrasive water jet machined surface has a characteristic appearance with more or less pronounced striations. Looking at a cross-section of the machined surface, the striations define its waviness. As the depth of the cut and traverse speed increases, the striations are more pronounced.

The output from each production process is a machined part. Accuracy and machining quality are most often used to estimate process quality, and the same can be applied to abrasive water jet machining. These metrics are the main limitations to applying this technique more widely, which is why it is very important to understand the process of abrasive water jet machined surface formation and its topography. Striations define the roughness and waviness of the machined surface in the lower cut zone, and explaining the cause and mechanism of their formation can help in achieving better quality machined surfaces.

There are numerous papers dealing with the influence of abrasive water jet machining parameters on machined surface roughness. In practice, it is easiest to change the traverse speed on an abrasive water jet machine, which is why most authors focus on the influence of traverse speed on quality, i.e., the roughness of the machined surface [4,5]. A significantly smaller number of authors deal with machined surface waviness, as well as the parameters that affect it, in their research.

The appearance of surfaces machined with abrasive water jets and striation formation are best described—and in most detail—in the works of Hashish [6,7,8]. By cutting transparent materials and recording the movement of an abrasive water jet through them, Hashish described the machined surface creation process. By cutting different materials with variable operating pressures, he analyzed the influence of this parameter on the maximum depth of cut, the traverse speed at which the material can be completely cut, and waviness height. Among other things, he determined that, for the same traverse speed values, but when machining with lower operating pressure values, the waviness of the machined surface is more pronounced than with higher pressure values. Guo [9] et al. analyzed abrasive water jet cutting in three dimensions. The topography of the machined surface was analyzed using Fourier transformation. They found that, during cutting, the abrasive water jet oscillates unsteadily and perpendicular to the traverse speed direction, in the rough cutting region. In the lower part of the cut, striations and grooves are more pronounced. The wave length of these grooves and striations depends on the diameter of the abrasive water jet and the abrasive mash size. Plodzien et al. [10] investigated the influence of traverse speed, abrasive mass flow rate and thickness on kerf angle, surface roughness and waviness on Inconel 718 alloy. They concluded that the surface roughness and waviness of a machined surface varies with cut depth, smoothly without a clear boundary between smooth and rough regions. However, the thickness of the sample has no impact on the roughness and waviness at a particular depth of cut. Surface waviness is equally influenced by depth of cut and traverse speed, but abrasive mass flow has a negligible effect. Chao and Geskin [11] investigated surface topography formation during abrasive water jet cutting by performing a spectral analysis of the machined surface topography They determined that the amplitude of striation mark amplitude is a second-degree polynomial function of the depth of cut, as well as a function of traverse speed. Also, they found that the cutting direction significantly affects striation appearance.

There are few studies that investigate pressure oscillations and their influence on striation appearance. Chalmes [12] analyzed operating pressure oscillation values and the influence of attenuator size thereupon. It has been determined that the attenuator volume affects the operating pressure oscillation value. When an attenuator with a 2130 mL capacity is used with a single intensifier pump, the working pressure values deviate by 2.5%. The larger dual intensifiers have higher operating pressure oscillation values (up to 5.4%). In their research, Trieb, Karl and Moderer [13] investigated pressure and flow rate fluctuations at intensifier pumps. Their paper compares the hydraulic pressure inside the actuating cylinder at the intensifier with the high pressure level and flow rate on the pump’s discharge connection. They determined that, by using the attenuator, the operating pressure oscillation values can be reduced from 150 MPa to 25 MPa. Their research was carried out on an intensifier pump whose characteristics were a maximum operating pressure of 400 MPa, a water flow of 3.6 L/min and the number of intensifier strokes of 32 min^−1^. In their paper, Xu, You and Kong [14] developed and described a phased intensifier system, which consists of a constant pressure variable capacity pump that provides compressed air to push the intensifier’s piston back, as well as a PLC to control the two pistons’ working sequence. Experimental investigations showed that the operating pressure fluctuation rate is no more than 2.0%. It also provides a good cutting quality and high production efficiency. Monno and Ravasio [15] analyzed the effect of pressure on pure water jet cutting. For this, rubber and polycarbonate samples were cut with a water jet at different operating pressure values. No correlation has been found between roughness profiles and operating pressure signal. The striations along a machined surface presuppose that there are other factors that directly influence its topography.

## 2. Abrasive Water Jet Machining

Modern abrasive water jet machines consist of a driver, as well as executive and supporting components. The executive component is a cutting head, while the driver is a unit that creates high-pressure water.

**Figure 1:**
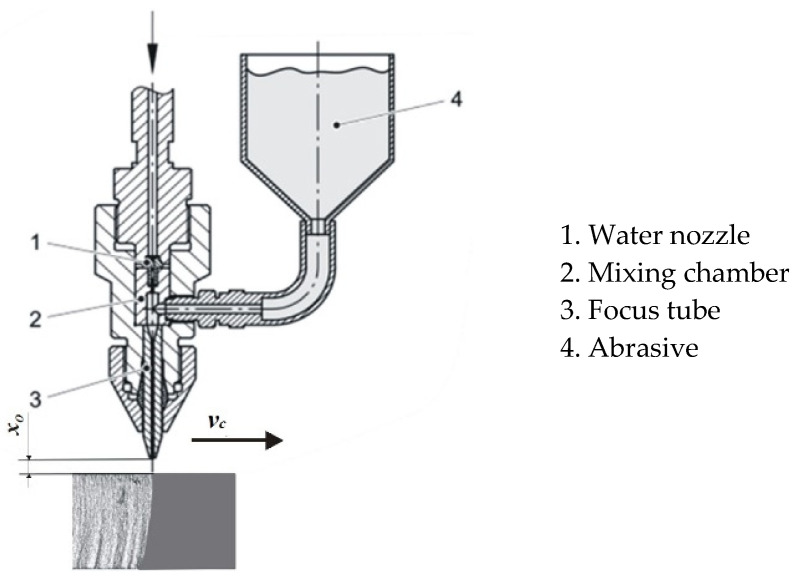
Abrasive water jet [16].

The cutting head is the part where the high-pressure water mixes with the abrasive and where the abrasive water jet is created. A schematic representation of the cutting head is given in Figure 1.

In abrasive water jet machining, a very-high-speed and high-pressure jet is used as a tool. The water that enters the cutting head—that is, the water nozzle—is usually under a pressure of 130 MPa to 600 MPa. The diameter of the water nozzle ranges from 0.08 mm to 0.4 mm. Due to this small diameter, the water jet reaches very high speeds of up to 1400 m/s. This jet further reaches the mixing chamber, where it is mixed with abrasive particles. The water jet accelerates the abrasive particles and together they pass through the long cylindrical focus tube. The mixture then exits this tube as a coherent abrasive water jet and performs machining.

**Figure 2:**
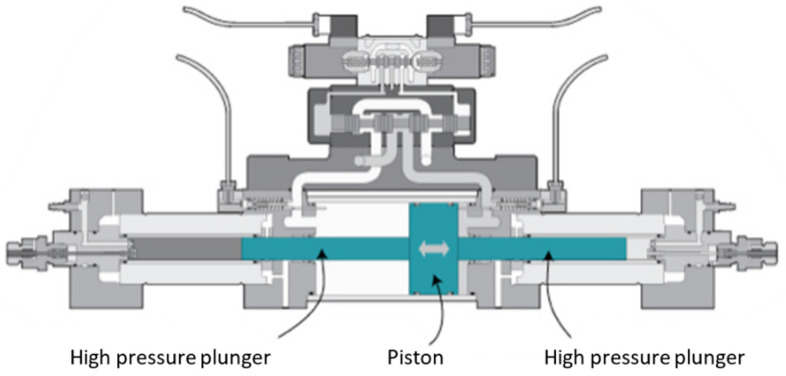
Intensifier [17].

Figure 2 shows the intensifier, which is the driver unit of the abrasive water jet machine. The working principle is as follows: Hydraulic oil under pressure of 5 MPa up to 35 MPa enters the hydraulic cylinder and acts on the piston of the intensifier’s piston. Because of the large difference in the diameters of the high-pressure plunger and piston in the intensifier, the water pressure reaches 400 MPa or more. The intensifier is where the high-pressure water jet is created. The pressure value in intensifier depends on the cross-section ratio of the high-pressure plunger and piston [17].

**Figure 3:**
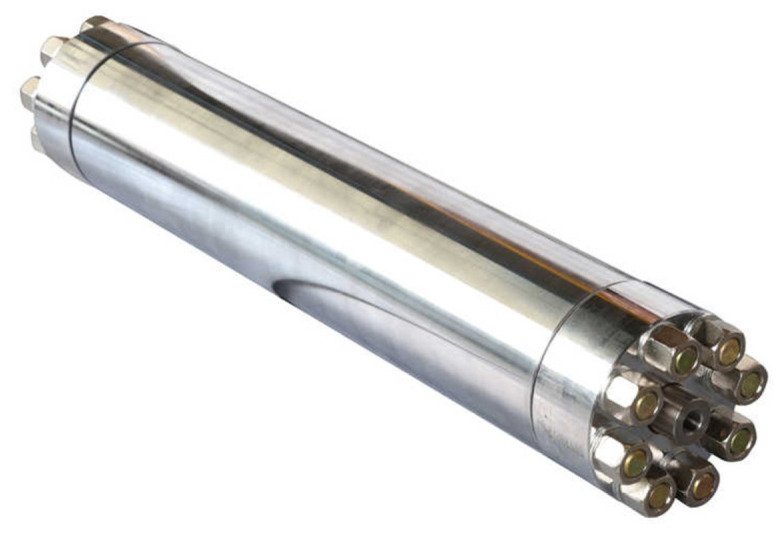
Pressure accumulator–attenuator [18].

When one high-pressure plunger completes its stroke, the second begins and compresses the water in front of it to the set pressure. At a pressure of 420 MPa, water can be compressed by 12% [14]; the first part of the working stroke of the high-pressure cylinder is thus used to compress the water, which means that there is a pressure drop in that interval. These pressure oscillations can range up to 150 MPa. For this reason, a pressure accumulator–attenuator is installed at the outlet of the intensifier, as shown in Figure 3.

### Abrasive Water Jet Machined Surface

**Figure 4:**
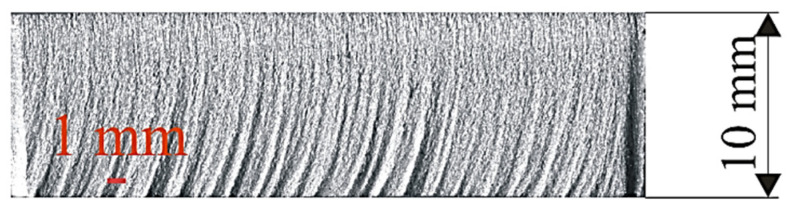
Appearance of a surface machined with an abrasive water jet.

Surfaces machined with an abrasive water jet have a characteristic appearance, as can be seen in Figure 4. Striations can be observed on the machined surface, which are characteristic of all treatments with a high-energy beam. These striations show the movement of the abrasive water jet through the workpiece material, determining the waviness of the machined surface. The waviness and roughness are the most important macroscopic properties of a surface machined with an abrasive water jet.

The method of creating a machined surface, as well as the influence of certain machining process parameters on its appearance, was first explained by Hashish [19]. The influence of the traverse speed, operating pressure and abrasive mass flow rate are most often investigated. Abrasive water jet machined surface can be divided into two zones: the fine machining (the upper zone) and rough zones (the lower zone). Roughness increases with the depth of cut (or thickness) of the material. Also, the appearance of striations is more pronounced. These striations define the waviness of the machined surface in the lower zone.

## 3. Materials and Methods

The purpose of this study was to determine whether there is a relationship between oscillations in operating pressure values and machined surface topography. The basic assumption is that oscillations in hydraulic oil pressure values at the output of the hydraulic pump are linked to water pressure–operating pressure value oscillations, and that the pressure attenuator does not completely eliminate them latter.

**Figure 5:**
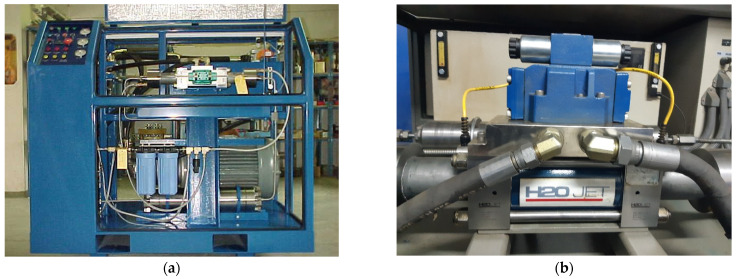
The driver unit (**a**) and intensifier (**b**) of an abrasive water jet cutting machine.

Experimental tests were performed on a PTV-3.8/60 abrasive water jet machine (PTV, Hostivice, Czech Republic) with a KMT cutting head. The driving part of the machine, as shown in Figure 5a, is equipped with a H2O Jet intensifier, with a maximum pressure of 413 MPa (Figure 5b). The number of intensifier strokes during the experiments was approximately 90 min^−1^.

The machining process parameters were kept constant during the AWJ cutting of the samples: the operating pressure was 413 MPa, the abrasive mass flow rate was 350 g/min, the standoff distance *x_o_* was 3 mm and the abrasive garnet mesh size was 80. The water nozzle (orifice) diameter was 0.3 mm and the focusing tube diameter was 1.02 mm. Only the traverse speed was varied.

**Figure 6:**
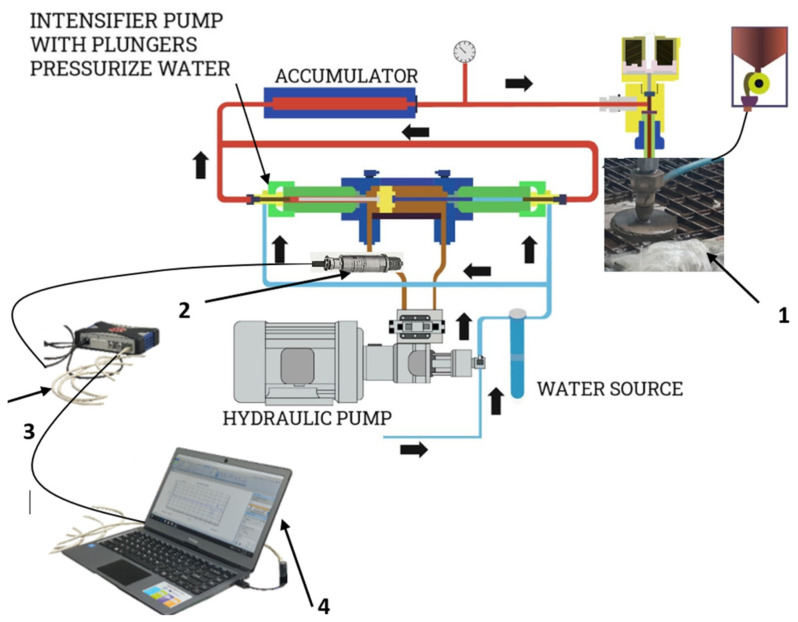
The experimental setup for measuring operating pressure pulsation.

**Figure 7:**
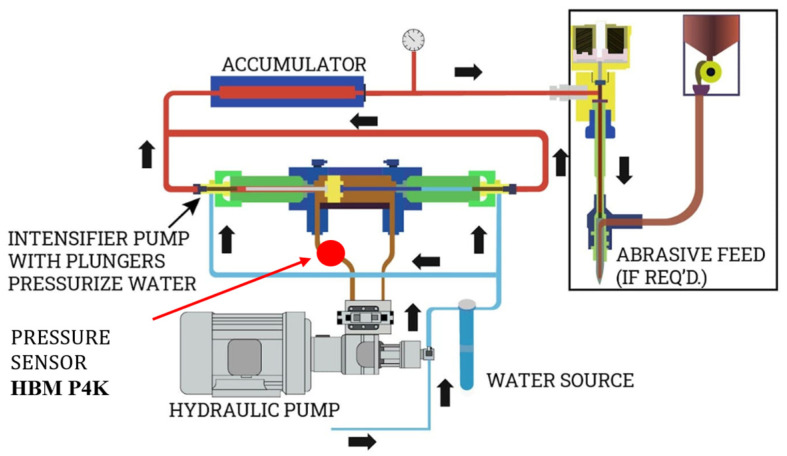
The system components of an abrasive water jet cutting machine and pressure sensor position [20].

The experimental setup for measuring operating pressure pulsation is based on a PC, as shown in Figure 6. It consists of the following equipment: (1) the test sample, (2) a pressure sensor HBM P4K (for pressures up to 50 MPa), (3) an HBM QuantumX MX80 digital amplifier and (4) a PC with LabVIEW 2008 software. The pressure sensor was mounted at the pump’s hydraulic oil output. The test samples are aluminum bars (50 mm wide and 700 mm long). Aluminum is of ENAW-5754 (AlMg3) quality, and the main alloying element is magnesium. The tensile strength of AlMg3 is in the range of 80 MPa to 280 MPa and the Brinell Hardness (HB) ranges from 52 to 88. The bars were 6 mm, 8 mm, 10 mm and 12 mm thick. Samples were cut with different traverse speeds: 200 mm/min, 400 mm/min, 600 mm/min, 800 mm/min, 1000 mm/min and 1200 mm/min. During sample cutting, the pressure at the hydraulic pump exit was measured, as shown in Figure 7. The HBM P4K pressure transmitter was used for these measurements.

## 4. Results and Discussion

**Figure 8:**
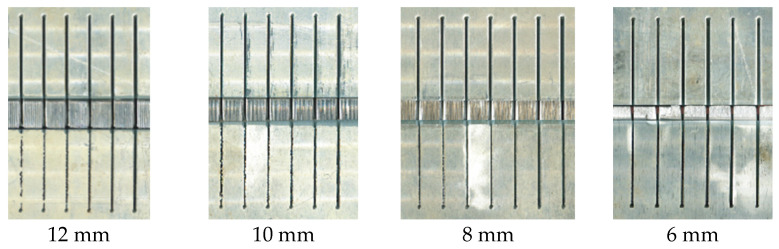
Samples.

For the experimental research carried out, AlMg3 was cut at different thicknesses: 6 mm, 8 mm, 10 mm and 12 mm. Each test sample contained six cuts, made with different traverse speeds: 200 mm/min, 400 mm/min, 600 mm/min, 800 mm/min, 1000 mm/min and 1200 mm/min. The cuts were 35 mm long, and the rods were not cut all the way. The samples obtained during the experimental research are shown in Figure 8. The samples were then cut completely using the EDM process in order to examine the machined surface.

Hydraulic oil pressure was monitored for each cut. The obtained signals were analyzed in detail and compared with the corresponding sample surfaces.

**Figure 9:**
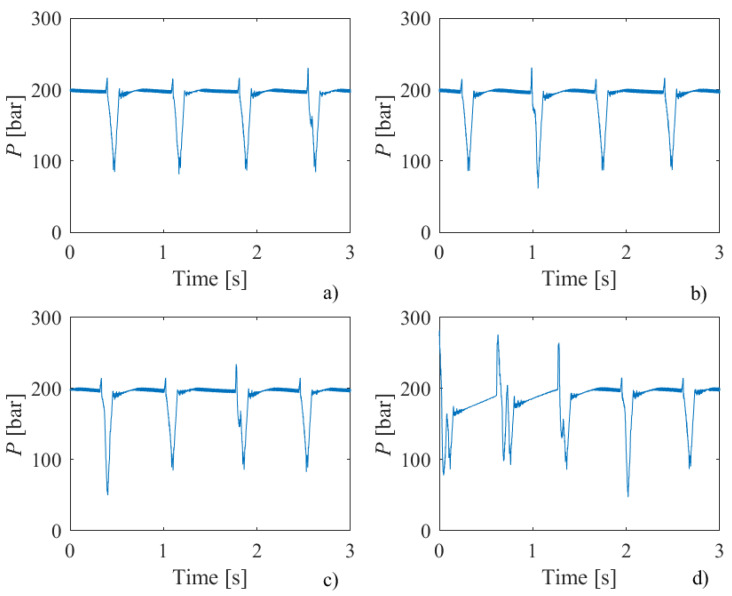
Hydraulic oil pressure diagrams for samples of different thicknesses—(**a**) 6 mm, (**b**) 8 mm, (**c**) 10 mm, (**d**) 12 mm—and a traverse speed of 200 mm/min.

The purpose of the research conducted here was to determine whether there is a connection between oscillations in operating pressure values and machined surface topography. In the intensifier, operating pressure is created by multiplying the hydraulic oil pressure. As already pointed out, the basic assumption is that the attenuator–pressure accumulator does not completely eliminate oscillations in the operating pressure value. Figure 9 shows hydraulic oil pressure diagrams when cutting samples of different thicknesses at a traverse speed of 200 mm/min.

From the diagrams shown in Figure 9, it can be observed that oscillations in pressure values occur at almost regular time intervals. By analyzing the time differences in the appearance of pressure peaks, it can be concluded that the number of peaks corresponds to the number of intensifier strokes (90 strokes/min). This means that the appearance of peaks in the pressure diagrams is determined by the intensifier’s operation mode. This regularity in the appearance of pressure pulsations was also observed in samples machined with higher traverse speeds.

**Figure 10:**
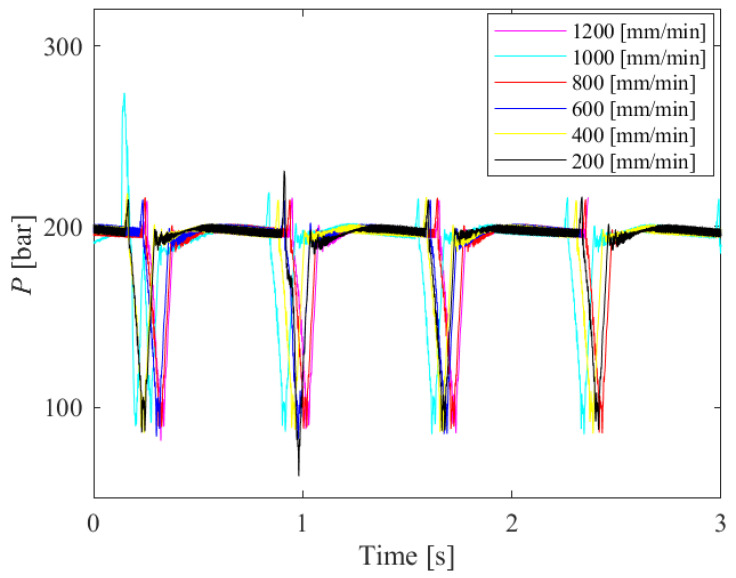
Hydraulic oil pressure peak matching diagram for 8 mm thick samples, cut with all traverse speed values.

No direct connection was observed when comparing the pressure diagram and the corresponding machined sample surface. After that, the coincidence of the pressure peaks when cutting samples at other traverse speeds was checked. Figure 10 shows the matching diagram of hydraulic oil pressure peaks for 8 mm thick samples, cut with all traverse speed values. It can be seen from the diagram that the peaks of the pressure values coincide regardless of traverse speed.

**Figure 11:**
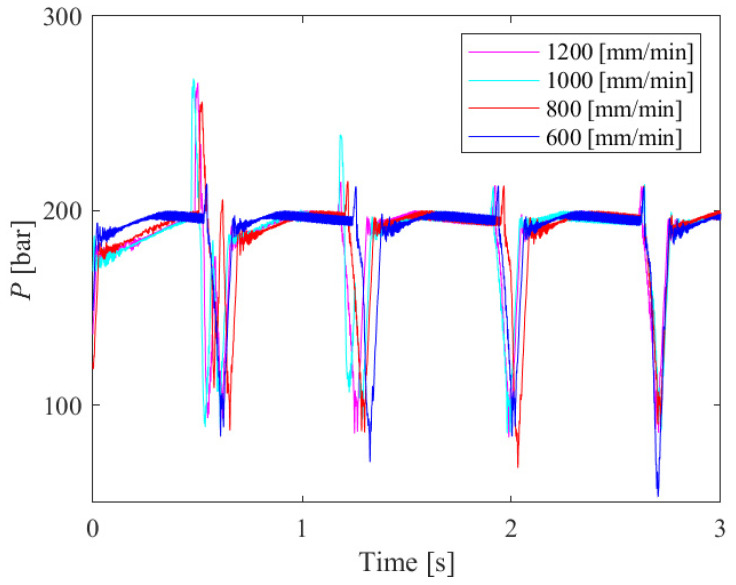
Hydraulic oil pressure diagrams for a 12 mm thick sample at traverse speeds of 600, 800, 1000 and 1200 mm/min.

When cutting samples of small thicknesses (6 mm and 8 mm) or cutting with a low traverse speed (200 mm/min and 400 mm/min), we observed that the machined surfaces have a good quality with no pronounced striations or other irregularities. For this reason, 10 mm and 12 mm thick samples obtained by cutting at traverse speeds of 600 to 1200 mm/min were analyzed. Figure 11 shows the hydraulic oil pressure diagram for a 12 mm thick sample.

The diagram shows that the pressure peaks match quite well; however, when these diagrams are compared with the corresponding machined surfaces, no matches can be observed.

Most devices with rotary motion contain various components for transforming power and motion, i.e., gears, belts, shafts, bearings, pump impellers, etc. The noise and vibration signals that resulting from the movement of all these components are directly related to the revolution speed of the shaft connected to the rotary parts. For example, the condition of rotating machinery can be monitored by measuring and analyzing these signals at certain locations [21]. The device considered in this paper is a mechanical system for transforming the rotary motion energy of an electric motor into the energy of a water jet and abrasive particles, both of which impact cutting. Therefore, the response of such a system under periodic loading due to rotary operation should respond, in terms of pressure, as a superposition of sinusoids whose frequencies are integer or fractional integer multiples of the loading revolution speed. Therefore, we can expect that striations will periodically appear on the machined surface of samples at intervals (periods) corresponding to the sinusoidal component frequencies contained in the pressure signal *P*(*t*), where *t* is time.

**Figure 12:**
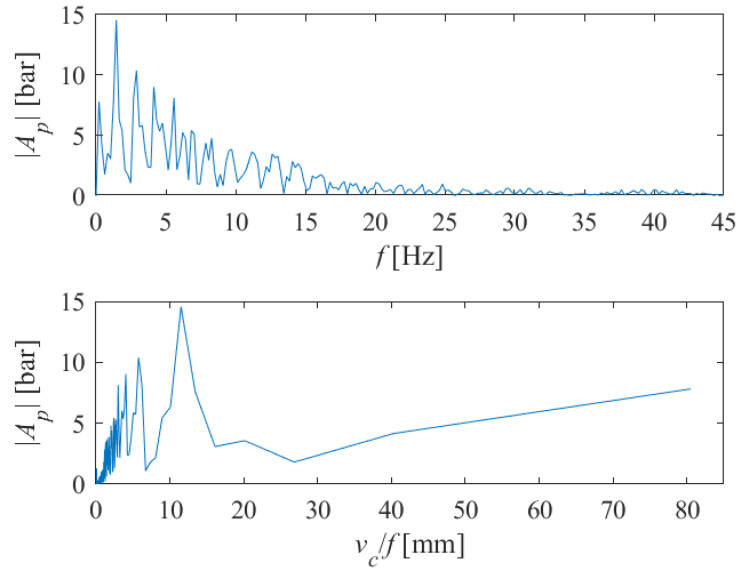
The single-sided amplitude spectrum of *P*(*t*) versus frequencies (**above**), and versus periods (**below**) at which the appearance of striations on the machined surface can be expected (*v_c_* = 1000 mm/min, *s* = 10 mm).

By applying discrete Fourier transform (DFT) of the pressure signal using a fast Fourier transform (FFT) algorithm in Matlab, we obtained the single-sided amplitude spectrum of *P*(*t*), as shown in Figure 12 (above). Here, |*A_p_*| denotes the complex magnitude of the spectrum and *f* is a frequency. If we divide the traverse speed *v_c_* [mm/s] by the instantaneous frequency *f* [Hz], we will obtain the transformed single-sided amplitude spectrum of *P*(*t*) versus periods where striations can be expected to appear on the machined surface (in millimeters). This can be seen in Figure 12 (below).

**Figure 13:**
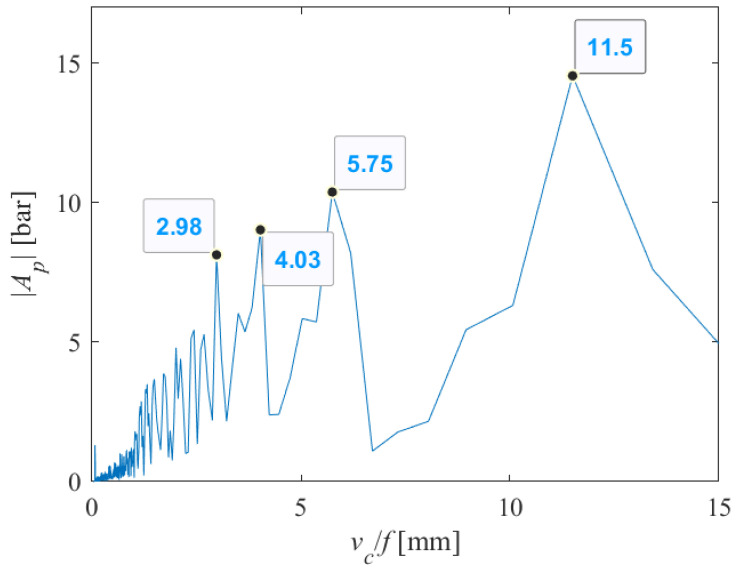
Part of the single-sided amplitude spectrum of *P*(*t*) versus periods at which the appearance of striations on the machined surface can be expected (*v_c_* = 1000 mm/min, *s* = 10 mm).

The length of the samples on which the tests were performed was 35 mm. Since side effect-induced non-stationary conditions occur at the end and beginning of the abrasive water jet cutting process, we excluded all spectral components whose period was greater than about twenty millimeters. For the case shown in Figure 12, it is sufficient to analyze the spectral content shown in Figure 13, in the range of up to 15 mm.

From Figure 13, we can see that many harmonics appear, with those with periods of 2.98, 4.03, 5.75 and 11.5 carrying the greatest energy. Let us assume that 4*x* = 11.5; then, 2*x* = 5.75 and 1.04*x* = 2.98. Therefore, two integer and one rational multiples appear, very close to the fundamental harmonic, though the harmonic with an amplitude 1.4*x* = 4.03 remains dominant.

In condition monitoring for rotating machines and equipment, this phenomenon is well known. More specifically, when the change in the drive shaft speed during measurement (pressure, in this case) is known, rational harmonics can be directly linked to gear pairs or other components in the rotating system under analysis [21,22]. Here, when measuring pressure, the speed of the drive shaft was not measured in parallel, so it is not possible to directly link the present harmonics to the system’s physical components.

**Figure 14:**
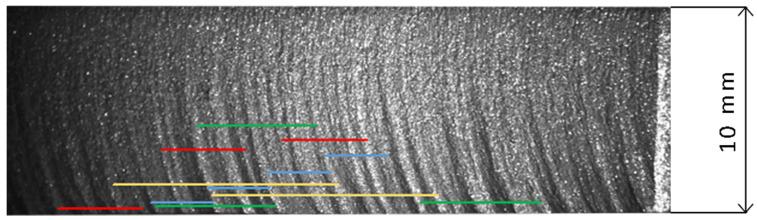
Visual pattern of surface related to dominant harmonics in pressure signal (2.98 blue, 4.03 red, 5.75 green, 11.5 yellow) (*v_c_* = 1000 mm/min, *s* = 10 mm).

Figure 14 shows the identified coincidences of striation intervals on the machined surface of the sample with the previously identified maximum amplitude harmonics. It can be seen that all four harmonics are clearly visible on the surface and that they appear multiple times. Of course, the actual appearance of the machined surface of the sample is a complex superposition of all harmonics present in the signal.

**Figure 15:**
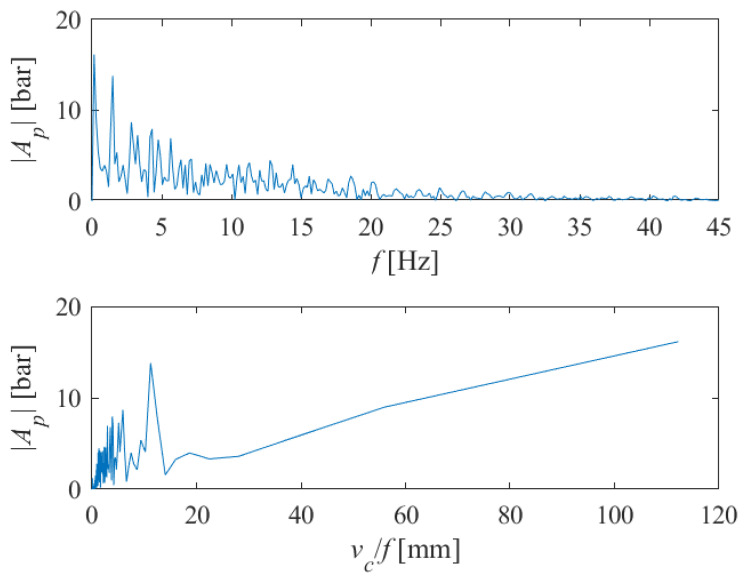
The single-sided amplitude spectrum of *P*(*t*) versus frequencies (**above**), and versus periods (**below**) at which the appearance of striations on the machined surface can be expected (*v_c_* = 1000 mm/min, *s* = 12 mm).

**Figure 16:**
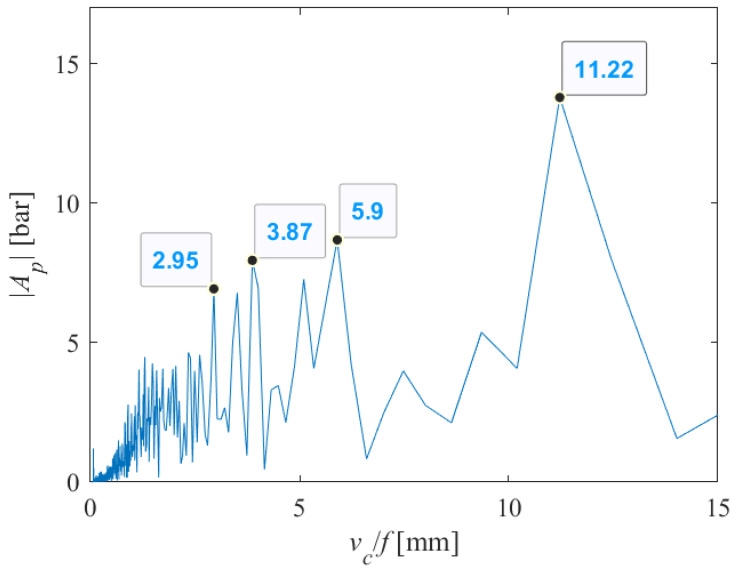
Part of the single-sided amplitude spectrum of *P*(*t*) versus periods at which the appearance of striations on the machined surface can be expected (*v_c_* = 1000 mm/min, *s* = 12 mm).

**Figure 17:**
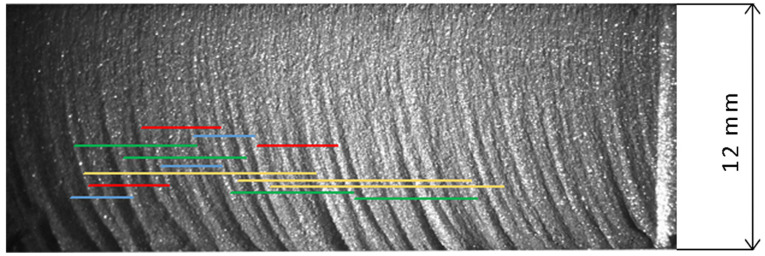
Visual pattern of surface related to dominant harmonics in pressure signal (2.95 blue, 3.87 red, 5.9 green, 11.22 yellow) (*v_c_* = 1000 mm/min, *s* = 12 mm).

The analysis was conducted in the same way for the case when *v_c_* = 1000 mm/min, *s* = 12 mm. The obtained results are shown in Figure 15, Figure 16 and Figure 17. Here, the harmonics *x* = 2.95, 1.3*x* = 3.87, 2*x* = 5.9 and 3.8*x* = 11.22 appear, as the most easily noticeable on the machined surface.

## 5. Conclusions

Starting from the electric drive, through the pump and all other rotating parts of the machine, the AWJ machine system itself, and the machined material have a rotational motion that transforms power and motion. It is widely known that the frequencies of noise and vibration signals that occur are directly related to the revolution speed of the shaft connected to the rotary parts. For example, the condition of rotating machinery can be monitored by measuring and analyzing these signals at certain locations [21,22].

Analogously, we can expect that when the energy of the rotary motion of the electric motor is transformed into abrasive water jet energy, periodic loading due to rotary operation occurs. Responding pressure measurements are sinusoid superpositions whose frequencies are integer or fractional integer multiples of the loading revolution speed. As a result of periodic loading, striations should appear periodically on the machined surface of the workpiece at intervals (periods) directly proportional to the sinusoidal component (harmonic) frequencies contained in the pressure signal. By applying the fast Fourier transform (FFT) algorithm, the single-sided pressure signal amplitude spectrum can be found. By multiplying inverse frequency data with the traverse speed, the transformed single-sided amplitude spectrum against the period in which the appearance of striations is expected (in millimeters) can be obtained.

As we have shown in several examples, in the lower zone of a machined surface, where stationary cutting conditions occur, striations are readily observed at intervals determined by dominant harmonics. This means that the machined surface topography is directly induced by the frequency characteristics of the water jet pressure.

The relationship between the harmonic content of the pressure signal and the appearance of striations on the cut surface topography can be used in designing AWJ machines, especially for optimizing drive systems. For example, designers might incorporate a comparative analysis of the harmonic characteristics of hydraulic pumps and intensifiers that are applied—or that might potentially be applied—to AWJ machines. Comparative experimental tests on machines of different types, powers, and characteristics can be used to form an experimental database, which could provide designers with significant information for further improving the characteristics of abrasive water jet machines in terms of machined surface waviness.

Future research includes research on establishing a functional dependence between the harmonic content of the pressure signal and the appearance of striations on the cut surface topography. For a more detailed analysis of the causes of oscillations in abrasive water jet systems, additional experimental tests are required. In such tests, the angular position of the electric motor drive rotor should be detected, in addition to the pressure, because in non-stationary conditions, with larger oscillations in the number of drive machine revolutions, close harmonic overlapping and masking occur during standard frequency analysis [21,22]. Simultaneous measurement of the angular position of the drive system shafts and hydraulic oil pressure, in order to accurately see whether, and to what extent, close or crossing harmonics appear in the hydraulic oil pressure signal, is required.

Also, other machining parameters should be varied in order to be able to develop a reliable model that defines the connection between the harmonic content of the pressure signal and the appearance of striations on the cut surface topography.

## Data Availability

The original contributions presented in this study are included in the article. Further inquiries can be directed to the corresponding authors.
